# Di-μ-chlorido-bis­[bis­(ethyl­enediamine-κ^2^ *N*,*N*′)cadmium(II)] dichloride

**DOI:** 10.1107/S1600536809054804

**Published:** 2009-12-24

**Authors:** Christian Näther, Inke Jess

**Affiliations:** a Institut für Anorganische Chemie, Christian-Albrechts-Universität Kiel, Olshausenstrasse 40, D-24098 Kiel, Germany

## Abstract

The crystal structure of the title compound, [Cd_2_Cl_2_(C_2_H_8_N_2_)_4_]Cl_2_, consists of binuclear centrosymmetric [Cd_2_(C_2_H_8_N_2_)_4_Cl_2_]^2+^ cations and discrete chloride anions. The Cd^II^ cation is coordinated by four N atoms of two ethyl­enediamine ligands and two symmetry-related chloride anions within a distorted CdN_4_Cl_2_ octa­hedron. Two Cd^II^ cations are connected by two chloride anions *via* μ_2_-coordination, forming a four-membered Cd_2_Cl_2_ ring. The uncoordinated chloride anions are linked to the amino groups *via* N—H⋯Cl hydrogen bonding. Two C atoms of one of the two crystallographically independent ethyl­enediamine ligands are disordered and were refined using a split model [occupancy ratio 0.674 (9):0.326 (9)].

## Related literature

For the general background to this work see: Bhosekar *et al.* (2006 ▶); Näther *et al.* (2007**a* ▶,b* ▶). For related structures, see: Cannas *et al.* (1980 ▶); Marsh (1999 ▶); Pauly *et al.* (2000 ▶); Chen *et al.* (2005 ▶).
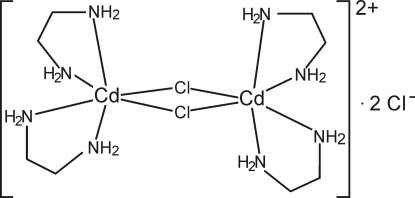


## Experimental

### 

#### Crystal data

[Cd_2_Cl_2_(C_2_H_8_N_2_)_4_]Cl_2_
*M* *_r_* = 607.02
Monoclinic, 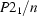

*a* = 6.3869 (8) Å
*b* = 11.3143 (10) Å
*c* = 14.8255 (19) Å
β = 92.621 (13)°
*V* = 1070.2 (2) Å^3^
*Z* = 2
Mo *K*α radiation
μ = 2.49 mm^−1^
*T* = 293 K
0.3 × 0.2 × 0.2 mm

#### Data collection

Stoe IPDS-1 diffractometer
Absorption correction: numerical (*X-SHAPE*; Stoe & Cie, 1998 ▶) *T*_min_ = 0.576, *T*_max_ = 0.613
6562 measured reflections
3110 independent reflections
2699 reflections with *I* > 2σ(*I*)
*R*_int_ = 0.018

#### Refinement

*R*[*F* ^2^ > 2σ(*F* ^2^)] = 0.018
*wR*(*F* ^2^) = 0.047
*S* = 1.04
3110 reflections
120 parameters
H-atom parameters constrained
Δρ_max_ = 0.53 e Å^−3^
Δρ_min_ = −0.49 e Å^−3^

### 

Data collection: *DIF4* (Stoe & Cie, 1992 ▶); cell refinement: *DIF4*; data reduction: *REDU4* (Stoe & Cie, 1992 ▶); program(s) used to solve structure: *SHELXS97* (Sheldrick, 2008 ▶); program(s) used to refine structure: *SHELXL97* (Sheldrick, 2008 ▶); molecular graphics: *XP* in *SHELXTL* (Sheldrick, 2008 ▶); software used to prepare material for publication: *XCIF* in *SHELXTL*.

## Supplementary Material

Crystal structure: contains datablocks I, global. DOI: 10.1107/S1600536809054804/wm2291sup1.cif

Structure factors: contains datablocks I. DOI: 10.1107/S1600536809054804/wm2291Isup2.hkl

Additional supplementary materials:  crystallographic information; 3D view; checkCIF report

## Figures and Tables

**Table 1:** Selected bond lengths (Å)

Cd1—N2	2.3268 (15)
Cd1—N3	2.3314 (15)
Cd1—N1	2.3513 (14)
Cd1—N4	2.3971 (16)
Cd1—Cl1^i^	2.6200 (5)
Cd1—Cl1	2.7078 (5)

Symmetry code: (i) 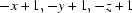.

**Table 2:** Hydrogen-bond geometry (Å, °)

*D*—H⋯*A*	*D*—H	H⋯*A*	*D*⋯*A*	*D*—H⋯*A*
N1—H1*N*1⋯Cl2^ii^	0.90	2.73	3.6137 (15)	168
N1—H2*N*1⋯Cl2^i^	0.90	2.54	3.3941 (15)	159
N2—H2*N*2⋯Cl2^iii^	0.90	2.42	3.3123 (15)	171
N3—H1*N*3⋯Cl1^iv^	0.90	2.53	3.3581 (16)	154
N3—H2*N*3⋯Cl2	0.90	2.67	3.4919 (17)	152
N4—H3*N*4⋯Cl2^ii^	0.90	2.78	3.653 (2)	164
N4—H4*N*4⋯Cl2^iii^	0.90	2.85	3.708 (2)	161

Symmetry codes: (i) 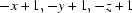; (ii) 
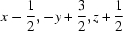
; (iii) 
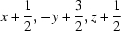
; (iv) 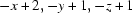.

**Fig. 1.**
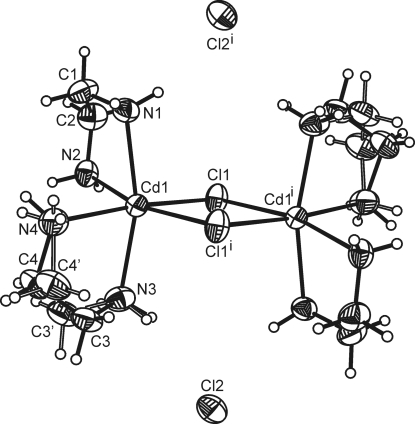
: Crystal structure of the title compound with labelling and displacement ellipsoids drawn at the 50% probability level. Symmetry code: i=-x + 1, -y + 1, -z + 1.

## supplementary crystallographic information

### Comment

Recently, we became interested in the synthesis, structures and thermal behaviour of coordination polymers based on zinc(II) halides and *N*-donor ligands. We have found out that new ligand-deficient coordination polymers can simply be prepared by thermal decomposition of suitable ligand-rich precursor compounds (Bhosekar *et al.*, 2006; Näther *et al.*, 2007*a*,*b*). In related studies we started to investigate the properties of the heavier homologue cadmium. As a part of this project the crystal structure of the title compound, [Cd_2_(C_2_H_8_N_2_)_4_Cl_2_]Cl_2_, was investigated.

In the crystal structure discrete [(C_2_H_8_N_2_)_4_Cd_2_Cl_2_]^2+^ cations are found which are located on centres of inversion. The structure contains additional chloride anions which are not connected to the cations and are located in general positions. In the complex cation the Cd^2+^ atoms are *cis*-coordinated by two symmetry related chloride anions and four N atoms of two crystallographically independent ethylenediamine ligands, leading to distorted CdN_4_Cl_2_ octahedra (Fig. 1 and Table 1). The Cd^2+^ atoms are linked by two symmetry-related chloride anions into a 4-membered centrosymmetric and planar Cd_2_Cl_2_ ring. This structural motif is known and found in some other Cd halide complexes (Cannas **et al.**, 1980; Marsh, 1999; Pauly *et al.*, 2000). Both Cd—Cl distances (Table 1) are different but comparable to those in the related compounds. The chloride anions that are not involved in Cd coordination are connected to the H atoms of the amino groups by N—H···Cl hydrogen bonding (Table 2).

### Experimental

0.1833 g CdCl_2_ (1 mmol) were reacted with 0.3005 g ethylenediamine (5 mmol) in a glass tube at room temperature. After three days colourless crystals of the title compound have formed as the minor phase in a mixture with the literature known compound tris(ethylenediamine-*N,N*')-cadmium(II) dichloride monohydrate (Chen *et al.*, 2005).

### Refinement

All H atoms were were positioned with idealized geometry and were refined isotropically with *U*_eq_(H) = 1.2 *U*_eq_(C,*N*) of the parent atom using a riding model with C—H = 0.97 and N—H = 0.90 Å. Two C atoms of one of the two crystallographically independent ethylenediamine ligands are disordered and were refined using a split model with a 0.674 (9): 0.326 (9) occupancy ratio.

### Crystal data

**Table:**

[Cd_2_Cl_2_(C_2_H_8_N_2_)_4_]Cl_2_	*F*(000) = 600
*M**_r_* = 607.02	*D*_x_ = 1.884 Mg m^−^^3^
Monoclinic, *P*2_1_/*n*	Mo *K*α radiation, λ = 0.71073 Å
Hall symbol: -P 2yn	Cell parameters from 120 reflections
*a* = 6.3869 (8) Å	θ = 10–25°
*b* = 11.3143 (10) Å	µ = 2.49 mm^−^^1^
*c* = 14.8255 (19) Å	*T* = 293 K
β = 92.621 (13)°	Block, colourless
*V* = 1070.2 (2) Å^3^	0.3 × 0.2 × 0.2 mm
*Z* = 2	

### Data collection

**Table:**

Stoe IPDS-1 diffractometer	3110 independent reflections
Radiation source: fine-focus sealed tube	2699 reflections with *I* > 2σ(*I*)
graphite	*R*_int_ = 0.018
φ scan	θ_max_ = 30.0°, θ_min_ = 2.3°
Absorption correction: numerical (*X-SHAPE*; Stoe & Cie, 1998)	*h* = 0→8
*T*_min_ = 0.576, *T*_max_ = 0.613	*k* = −15→15
6562 measured reflections	*l* = −20→20

### Refinement

**Table:**

Refinement on *F*^2^	Secondary atom site location: difference Fourier map
Least-squares matrix: full	Hydrogen site location: inferred from neighbouring sites
*R*[*F*^2^ > 2σ(*F*^2^)] = 0.018	H-atom parameters constrained
*wR*(*F*^2^) = 0.047	*w* = 1/[σ^2^(*F*_o_^2^) + (0.0256*P*)^2^ + 0.1639*P*] where *P* = (*F*_o_^2^ + 2*F*_c_^2^)/3
*S* = 1.04	(Δ/σ)_max_ = 0.002
3110 reflections	Δρ_max_ = 0.53 e Å^−^^3^
120 parameters	Δρ_min_ = −0.49 e Å^−^^3^
0 restraints	Extinction correction: *SHELXL97* (Sheldrick, 2008), Fc^*^=kFc[1+0.001xFc^2^λ^3^/sin(2θ)]^-1/4^
Primary atom site location: structure-invariant direct methods	Extinction coefficient: 0.0088 (4)

### Special details

**Table:**

Geometry. All e.s.d.'s (except the e.s.d. in the dihedral angle between two l.s. planes) are estimated using the full covariance matrix. The cell e.s.d.'s are taken into account individually in the estimation of e.s.d.'s in distances, angles and torsion angles; correlations between e.s.d.'s in cell parameters are only used when they are defined by crystal symmetry. An approximate (isotropic) treatment of cell e.s.d.'s is used for estimating e.s.d.'s involving l.s. planes.
Refinement. Refinement of *F*^2^ against ALL reflections. The weighted *R*-factor *wR* and goodness of fit *S* are based on *F*^2^, conventional *R*-factors *R* are based on *F*, with *F* set to zero for negative *F*^2^. The threshold expression of *F*^2^ > σ(*F*^2^) is used only for calculating *R*-factors(gt) *etc*. and is not relevant to the choice of reflections for refinement. *R*-factors based on *F*^2^ are statistically about twice as large as those based on *F*, and *R*- factors based on ALL data will be even larger.

### Fractional atomic coordinates and isotropic or equivalent isotropic displacement parameters (Å^2^)

**Table:**

	*x*	*y*	*z*	*U*_iso_*/*U*_eq_	Occ. (<1)
Cd1	0.658581 (17)	0.626379 (10)	0.561674 (7)	0.03352 (5)	
Cl1	0.70739 (6)	0.39404 (4)	0.52416 (3)	0.04109 (9)	
Cl2	0.67265 (8)	0.63867 (4)	0.20632 (4)	0.05032 (11)	
N1	0.5276 (2)	0.61258 (13)	0.70697 (10)	0.0382 (3)	
H1N1	0.4337	0.6703	0.7155	0.046*	
H2N1	0.4645	0.5423	0.7142	0.046*	
C1	0.7084 (3)	0.62488 (17)	0.77133 (11)	0.0475 (4)	
H1A	0.6709	0.5970	0.8303	0.057*	
H1B	0.7471	0.7076	0.7768	0.057*	
C2	0.8923 (3)	0.55455 (18)	0.73977 (13)	0.0491 (4)	
H2A	1.0072	0.5586	0.7848	0.059*	
H2B	0.8523	0.4723	0.7322	0.059*	
N2	0.9601 (2)	0.60183 (13)	0.65389 (10)	0.0394 (3)	
H1N2	1.0487	0.5512	0.6284	0.047*	
H2N2	1.0259	0.6715	0.6627	0.047*	
N3	0.8018 (2)	0.70351 (15)	0.43233 (10)	0.0447 (3)	
H1N3	0.9427	0.7019	0.4377	0.054*	0.326 (9)
H2N3	0.7598	0.6610	0.3835	0.054*	0.326 (9)
H3N3	0.9223	0.6660	0.4214	0.054*	0.674 (9)
H4N3	0.7121	0.6933	0.3844	0.054*	0.674 (9)
C3	0.7267 (15)	0.8259 (6)	0.4236 (4)	0.0468 (19)	0.326 (9)
H3C	0.7886	0.8641	0.3726	0.056*	0.326 (9)
H3D	0.5754	0.8271	0.4139	0.056*	0.326 (9)
C4	0.7884 (17)	0.8874 (6)	0.5075 (5)	0.054 (2)	0.326 (9)
H4C	0.7632	0.9716	0.5009	0.065*	0.326 (9)
H4D	0.9365	0.8753	0.5221	0.065*	0.326 (9)
C3'	0.8423 (7)	0.8322 (3)	0.4474 (3)	0.0536 (10)	0.674 (9)
H3A	0.8603	0.8708	0.3899	0.064*	0.674 (9)
H3B	0.9708	0.8421	0.4841	0.064*	0.674 (9)
C4'	0.6641 (8)	0.8893 (3)	0.4939 (3)	0.0557 (10)	0.674 (9)
H4A	0.6870	0.9738	0.4989	0.067*	0.674 (9)
H4B	0.5333	0.8760	0.4595	0.067*	0.674 (9)
N4	0.6547 (3)	0.83613 (14)	0.58432 (12)	0.0531 (4)	
H1N4	0.5228	0.8642	0.5798	0.064*	0.326 (9)
H2N4	0.7120	0.8550	0.6390	0.064*	0.326 (9)
H3N4	0.5368	0.8583	0.6107	0.064*	0.674 (9)
H4N4	0.7658	0.8588	0.6197	0.064*	0.674 (9)

### Atomic displacement parameters (Å^2^)

**Table:**

	*U*^11^	*U*^22^	*U*^33^	*U*^12^	*U*^13^	*U*^23^
Cd1	0.03410 (7)	0.03660 (7)	0.02973 (6)	−0.00457 (4)	0.00013 (4)	−0.00078 (4)
Cl1	0.02885 (16)	0.04039 (19)	0.0533 (2)	0.00407 (14)	−0.00646 (15)	−0.01037 (16)
Cl2	0.0504 (2)	0.0410 (2)	0.0597 (3)	0.00376 (17)	0.0042 (2)	0.01005 (18)
N1	0.0337 (6)	0.0439 (7)	0.0374 (6)	−0.0007 (5)	0.0052 (5)	0.0035 (5)
C1	0.0458 (9)	0.0665 (12)	0.0302 (7)	0.0017 (8)	0.0017 (6)	−0.0002 (7)
C2	0.0439 (9)	0.0571 (10)	0.0455 (9)	0.0089 (8)	−0.0067 (7)	0.0085 (8)
N2	0.0303 (6)	0.0422 (7)	0.0458 (7)	0.0000 (5)	0.0008 (5)	−0.0061 (6)
N3	0.0360 (7)	0.0607 (9)	0.0378 (7)	0.0050 (6)	0.0062 (5)	0.0027 (6)
C3	0.045 (4)	0.051 (3)	0.044 (3)	−0.003 (3)	0.002 (3)	0.019 (2)
C4	0.063 (5)	0.038 (3)	0.062 (4)	−0.008 (3)	0.008 (4)	0.013 (3)
C3'	0.047 (2)	0.0619 (19)	0.0529 (19)	−0.0065 (14)	0.0122 (16)	0.0177 (14)
C4'	0.059 (3)	0.0456 (15)	0.063 (2)	0.0064 (15)	0.0113 (17)	0.0172 (13)
N4	0.0788 (12)	0.0383 (7)	0.0430 (8)	0.0001 (8)	0.0106 (8)	−0.0019 (7)

### Geometric parameters (Å, °)

**Table:**

Cd1—N2	2.3268 (15)	N3—H1N3	0.9000
Cd1—N3	2.3314 (15)	N3—H2N3	0.9000
Cd1—N1	2.3513 (14)	N3—H3N3	0.9000
Cd1—N4	2.3971 (16)	N3—H4N3	0.9000
Cd1—Cl1^i^	2.6200 (5)	C3—C4	1.464 (12)
Cd1—Cl1	2.7078 (5)	C3—H3C	0.9700
Cl1—Cd1^i^	2.6200 (5)	C3—H3D	0.9700
N1—C1	1.471 (2)	C4—N4	1.566 (7)
N1—H1N1	0.9000	C4—H4C	0.9700
N1—H2N1	0.9000	C4—H4D	0.9700
C1—C2	1.511 (3)	C3'—C4'	1.503 (6)
C1—H1A	0.9700	C3'—H3A	0.9700
C1—H1B	0.9700	C3'—H3B	0.9700
C2—N2	1.465 (2)	C4'—N4	1.473 (4)
C2—H2A	0.9700	C4'—H4A	0.9700
C2—H2B	0.9700	C4'—H4B	0.9700
N2—H1N2	0.9000	N4—H1N4	0.9000
N2—H2N2	0.9000	N4—H2N4	0.9000
N3—C3	1.469 (7)	N4—H3N4	0.9000
N3—C3'	1.494 (4)	N4—H4N4	0.9000
			
N2—Cd1—N3	100.53 (6)	H1N3—N3—H3N3	31.4
N2—Cd1—N1	76.90 (5)	H2N3—N3—H3N3	80.1
N3—Cd1—N1	161.39 (5)	C3—N3—H4N3	81.8
N2—Cd1—N4	92.83 (6)	C3'—N3—H4N3	110.0
N3—Cd1—N4	75.61 (6)	Cd1—N3—H4N3	110.0
N1—Cd1—N4	86.05 (5)	H1N3—N3—H4N3	131.6
N2—Cd1—Cl1^i^	166.14 (4)	H2N3—N3—H4N3	30.7
N3—Cd1—Cl1^i^	90.48 (4)	H3N3—N3—H4N3	108.4
N1—Cd1—Cl1^i^	95.29 (4)	C4—C3—N3	107.4 (7)
N4—Cd1—Cl1^i^	98.09 (5)	C4—C3—H3C	110.2
N2—Cd1—Cl1	84.54 (4)	N3—C3—H3C	110.2
N3—Cd1—Cl1	98.12 (4)	C4—C3—H3D	110.2
N1—Cd1—Cl1	99.95 (4)	N3—C3—H3D	110.2
N4—Cd1—Cl1	172.69 (5)	H3C—C3—H3D	108.5
Cl1^i^—Cd1—Cl1	85.593 (13)	C3—C4—N4	107.9 (6)
Cd1^i^—Cl1—Cd1	94.407 (13)	C3—C4—H4C	110.1
C1—N1—Cd1	106.64 (10)	N4—C4—H4C	110.1
C1—N1—H1N1	110.4	C3—C4—H4D	110.1
Cd1—N1—H1N1	110.4	N4—C4—H4D	110.1
C1—N1—H2N1	110.4	H4C—C4—H4D	108.4
Cd1—N1—H2N1	110.4	N3—C3'—C4'	111.0 (3)
H1N1—N1—H2N1	108.6	N3—C3'—H3A	109.4
N1—C1—C2	110.35 (15)	C4'—C3'—H3A	109.4
N1—C1—H1A	109.6	N3—C3'—H3B	109.4
C2—C1—H1A	109.6	C4'—C3'—H3B	109.4
N1—C1—H1B	109.6	H3A—C3'—H3B	108.0
C2—C1—H1B	109.6	N4—C4'—C3'	107.7 (3)
H1A—C1—H1B	108.1	N4—C4'—H4A	110.2
N2—C2—C1	109.99 (14)	C3'—C4'—H4A	110.2
N2—C2—H2A	109.7	N4—C4'—H4B	110.2
C1—C2—H2A	109.7	C3'—C4'—H4B	110.2
N2—C2—H2B	109.7	H4A—C4'—H4B	108.5
C1—C2—H2B	109.7	C4'—N4—C4	30.7 (3)
H2A—C2—H2B	108.2	C4'—N4—Cd1	106.00 (17)
C2—N2—Cd1	106.55 (10)	C4—N4—Cd1	104.8 (3)
C2—N2—H1N2	110.4	C4'—N4—H1N4	82.3
Cd1—N2—H1N2	110.4	C4—N4—H1N4	110.8
C2—N2—H2N2	110.4	Cd1—N4—H1N4	110.8
Cd1—N2—H2N2	110.4	C4'—N4—H2N4	133.7
H1N2—N2—H2N2	108.6	C4—N4—H2N4	110.8
C3—N3—C3'	31.6 (3)	Cd1—N4—H2N4	110.8
C3—N3—Cd1	106.7 (2)	H1N4—N4—H2N4	108.9
C3'—N3—Cd1	108.26 (14)	C4'—N4—H3N4	110.5
C3—N3—H1N3	110.4	C4—N4—H3N4	135.0
C3'—N3—H1N3	80.9	Cd1—N4—H3N4	110.5
Cd1—N3—H1N3	110.4	H1N4—N4—H3N4	30.0
C3—N3—H2N3	110.4	H2N4—N4—H3N4	81.8
C3'—N3—H2N3	133.2	C4'—N4—H4N4	110.5
Cd1—N3—H2N3	110.4	C4—N4—H4N4	83.0
H1N3—N3—H2N3	108.6	Cd1—N4—H4N4	110.5
C3—N3—H3N3	135.0	H1N4—N4—H4N4	130.7
C3'—N3—H3N3	110.0	H2N4—N4—H4N4	29.5
Cd1—N3—H3N3	110.0	H3N4—N4—H4N4	108.7

Symmetry codes: (i) −*x*+1, −*y*+1, −*z*+1.

### Hydrogen-bond geometry (Å, °)

**Table:**

*D*—H···*A*	*D*—H	H···*A*	*D*···*A*	*D*—H···*A*
N1—H1N1···Cl2^ii^	0.90	2.73	3.6137 (15)	168
N1—H2N1···Cl2^i^	0.90	2.54	3.3941 (15)	159
N2—H2N2···Cl2^iii^	0.90	2.42	3.3123 (15)	171
N3—H1N3···Cl1^iv^	0.90	2.53	3.3581 (16)	154
N3—H2N3···Cl2	0.90	2.67	3.4919 (17)	152
N4—H3N4···Cl2^ii^	0.90	2.78	3.653 (2)	164
N4—H4N4···Cl2^iii^	0.90	2.85	3.708 (2)	161

Symmetry codes: (ii) *x*−1/2, −*y*+3/2, *z*+1/2; (i) −*x*+1, −*y*+1, −*z*+1; (iii) *x*+1/2, −*y*+3/2, *z*+1/2; (iv) −*x*+2, −*y*+1, −*z*+1.
